# A metabolome genome-wide association study implicates histidine *N*-pi-methyltransferase as a key enzyme in *N*-methylhistidine biosynthesis in *Arabidopsis thaliana*

**DOI:** 10.3389/fpls.2023.1201129

**Published:** 2023-06-08

**Authors:** Kai Uchida, June-Sik Kim, Muneo Sato, Hiromitsu Tabeta, Keiichi Mochida, Masami Yokota Hirai

**Affiliations:** ^1^ RIKEN Center for Sustainable Resource Science, Yokohama, Kanagawa, Japan; ^2^ Institute of Plant Science and Resources, Okayama University, Kurashiki, Okayama, Japan; ^3^ Kihara Institute for Biological Research, Yokohama City University, Yokohama, Kanagawa, Japan; ^4^ School of Information and Data Sciences, Nagasaki University, Nagasaki, Nagasaki, Japan; ^5^ RIKEN Baton Zone Program, Yokohama, Kanagawa, Japan; ^6^ Department of Applied Biosciences, Graduate School of Bioagricultural Science, Nagoya University, Nagoya, Japan

**Keywords:** GWAS, metabolomic analysis, *N*-methylhistidine, methyltransferase, LC-MS/MS

## Abstract

A genome-wide association study (GWAS), which uses information on single nucleotide polymorphisms (SNPs) from many accessions, has become a powerful approach to gene identification. A metabolome GWAS (mGWAS), which relies on phenotypic information based on metabolite accumulation, can identify genes that contribute to primary and secondary metabolite contents. In this study, we carried out a mGWAS using seed metabolomic data from *Arabidopsis thaliana* accessions obtained by liquid chromatography–mass spectrometry to identify SNPs highly associated with the contents of metabolites such as glucosinolates. These SNPs were present in genes known to be involved in glucosinolate biosynthesis, thus confirming the effectiveness of our analysis. We subsequently focused on SNPs detected in an unknown methyltransferase gene associated with *N*-methylhistidine content. Knockout and overexpression of *A. thaliana* lines of this gene had significantly decreased and increased *N*-methylhistidine contents, respectively. We confirmed that the overexpressing line exclusively accumulated histidine methylated at the pi position, not at the tau position. Our findings suggest that the identified methyltransferase gene encodes a key enzyme for *N*-methylhistidine biosynthesis in *A. thaliana*.

## Introduction

Plants accumulate a wide variety of metabolites. Plant-produced metabolites differ among species, and recent studies suggest that within-species metabolism is highly variable, both qualitatively and quantitatively, as well (Fernie and Tohge, 2017). To identify genes responsible for the variable accumulation of metabolites in plants, quantitative trait locus (QTL) analysis based on progenies of two parental strains that differ in a quantitative phenotype of interest has often been used. In recent years, genomes of representative accessions of single plant species as well as hundreds to thousands of accession-specific genomes have been determined, thereby providing a rich source of information on within-species genome-wide genetic variations, such as single nucleotide polymorphisms (SNPs). As a consequence, interest in population genomics has greatly increased.

A genome-wide association study (GWAS) is a method for identifying causative genetic loci of phenotypic variations through testing of genetic associations between genome-scale polymorphisms and phenotypic datasets. Compared with QTL mapping, which only assesses allelic diversity segregated between particular parents, the GWAS approach provides a higher resolution of causal coordinates and more fully explores phenotypic diversity in a natural population. As an extension, metabolic or metabolome GWAS (mGWAS), which uses metabolomic data for phenotypic information, has been applied to identify genes related to specific metabolites (Riedelsheimer et al., 2012; Angelovici et al., 2013; Chen et al., 2014; Matsuda et al., 2015; Slaten et al., 2020; Zeng et al., 2020; Wei et al., 2021; Zhao et al., 2023). Like a conventional QTL analysis, a GWAS can be used to find a causative gene of a specific phenotype of interest and thus serve as a hypothesis-driven approach to functional genomics. In addition, a GWAS can be applied as a data-driven approach, where a large number of phenotypes are first analyzed without setting any objective and a hypothesis is then formulated based on the results. Because it is not hypothesis-based, data-driven research has the potential to yield unexpected results. In fact, a number of cases have been reported in which GWASs have led to novel findings. For instance, a GWAS using osmotic tolerance as an indicator revealed the involvement of genes participating in pathogen resistance (Ariga et al., 2017). As another example, a study using glutamine-related traits in seeds revealed a trait association with aliphatic glucosinolate biosynthesis genes (Slaten et al., 2020).

In the present study, we performed a mGWAS using SNP information and metabolomic data from seeds of *Arabidopsis thaliana* accessions. This mGWAS revealed the association of many metabolites with SNPs. For example, the accumulation of glucosinolates, which are Brassicales-specific specialized metabolites, was associated with SNPs in known glucosinolate biosynthetic genes, thus validating our GWAS method. In many cases, however, functional characterization of genes showing associations with accumulations of some metabolites based solely on annotations was difficult. We subsequently focused on SNPs highly associated with the accumulation of pi-methyl histidine (πMH or 3-methyl-l-histidine) that were present in a gene (AT2G32160) annotated as a methyltransferase gene. Overexpression and knockout lines of this gene exhibited significantly increased and decreased πMH contents, respectively. This methyltransferase gene may thus be involved in the biosynthesis of πMH in *A*. *thaliana*. Our results demonstrate that data-driven mGWAS is a useful way to identify unexpected novel genes.

## Materials and methods

### Plants

Seeds of 245 *A. thaliana* accessions were purchased from the Arabidopsis Biological Resource Center. For the mGWAS, plants were grown from the original seeds in a greenhouse at 22°C under fluorescent light (16-h light/8-h dark), and mature seeds were harvested.

Seeds of SALK lines (Alonso et al., 2003) (AT2G32160, SALK_077267 and SALK_118137; AT2G32170, SALK_046329) (**Supplementary Figure 1**) were also purchased from the Arabidopsis Biological Resource Center. Homozygous mutants were confirmed by genomic PCR using primers obtained from T-DNA Express (http://signal.salk.edu/cgi-bin/tdnaexpress). The transgenic lines named *MT160*-OE and *MT170*-OE were established in the Col-0 background by introducing the CDS of AT2G32160.3 or AT2G32170.1 driven by the Cauliflower mosaic virus 35S promoter by the floral dip method (Clough and Bent, 1998) using *Agrobacterium tumefaciens* strain GV3101 (pMP90) transformed with vectors created as described below (under “Cloning”).

Transgenic plants were selected using GFP fluorescence as an indicator, and T_3_ seeds with confirmed fluorescence were used in subsequent experiments. Plants for the expression analysis were grown on half-strength Murashige and Skoog (MS) medium (10 g l^−1^ sucrose, 8 g l^−1^ agar, 1 g l^−1^ MES, and MS vitamin solution, pH 5.7) at 22°C under 16-h light/8-h dark conditions. After 3 weeks, aerial parts were sampled.

AT2G32160-OE plants for πMH analysis were cultured hydroponically as follows. Hydroponic sponges (2.5 × 2.5 × 2.5 cm; Kyowa, Osaka, Japan) were cut horizontally into thirds (2.5 × 2.5 × 0.8 cm), and a slit was introduced into each section. The divided sponges were then soaked in 1× Hyponica hydroponic liquid fertilizer (Kyowa). Water-absorbing sheets (Miki Tokushu Paper Mfg., Ehime, Japan) were soaked in the same medium. Roots of plants grown for 2 weeks under the above-mentioned conditions were placed between a half-folded absorbent sheet, placed in the sponge cutouts, and transferred to 50-ml amber conical tubes (Eppendorf, Hamburg, Germany) filled with the same liquid medium. The plants in each tube were lightly covered with plastic wrap and grown for 1 week. Col-0 plants were grown next to each OE plant as a control. Six grown plants each for different genotypes were used for the metabolomic analysis. A photograph of plants before sampling is shown in **Supplementary Figure 2**.

### Chemicals

πMH and tau-methyl-l-histidine (τMH) for GC-MS/MS structural analyses were purchased from Fujifilm Wako Pure Chemical Corporation (Osaka, Japan) as 3-methyl-l-histidine (product code 139-17851) and 1-methyl-l-histidine (product code 136-17861), respectively.

### Metabolomic analysis

To obtain metabolomic data for the mGWAS, seed samples were prepared by scooping approximately 50 seeds with a seed spoon (Bio Medical Science, Tokyo, Japan). Except for 10 accessions with insufficient seeds, prepared in triplicate, six replicates were prepared per accession. Metabolite extraction was performed according to previous studies (Sawada et al., 2009; Uchida et al., 2020). A metabolomic analysis for mGWAS was performed on a liquid chromatography–tandem quadruple mass spectrometry system (UPLC-TQS, Waters, Milford, MA, USA) according to previously reported methods (Sawada et al., 2009; Sawada et al., 2012) using the multiple reaction monitoring (MRM) conditions listed in **Supplementary Table 2**. The metabolome analysis included blank samples (only the extraction solvent) and the average intensity of the blank samples was used as the basal noise level. We generated six metabolomic datasets: three with data from 245 accessions, and three with data from 235 accessions.

For the metabolomic analysis, plant samples (aerial parts and roots) and seeds were suspended in 80% (v/v) methanol with 0.1% (v/v) formic acid and internal standards (8.4 nM of lidocaine and 210 nM of 10-camphorsulfonic acid) to a concentration of 4 mg/ml and approximately 50 seeds/ml, respectively, and extracted as described above. Quantification of πMH in plant samples and seeds of *MT160*- and *MT170*-OE and SALK lines was performed using LC-QqQ-MS (LCMS-8050, Shimadzu, Kyoto, Japan). The analysis was carried out according to our previous study (Uchida et al., 2020) using the MRM conditions detailed in **Supplementary Table 3**.

### mGWAS

We retrieved a publicly available, genome-wide polymorphism dataset of Arabidopsis accessions genotyped with the Arabidopsis 250k-SNP chip (Atwell et al., 2010) and generated a custom variation dataset comprising 213,925 biallelic SNP loci for these 220 accessions (**Supplementary Table 1**). To conduct a GWAS based on the six metabolome datasets, we used the multiple loci mixed linear model of GAPIT v3.1.0 (Wang and Zhang, 2021) in R v4.1.3. More specifically, we estimated the genetic relationship using a Multiple Loci Mixed Linear Model. Initially, we listed SNPs with *p*-value less than 1 × 10**^−^** ^5^ and from the coding regions of genes overlapping these SNPs, we complied 1,683 genes. We displayed the results as quantile–quantile and Manhattan plots using the R package qqman v0.1.8 (Turner, 2018). The in-house Python and R scripts used to build the custom dataset and perform the GWAS are available at a GitHub repository (https://github.com/junesk9/).

### Cloning

The coding sequence (CDS) of AT2G32160.3 was amplified by nested PCR using two sets of primers (first PCR: 5′-CTTCGTGTATACGAGGAACC-3′ and 5′-CTTAAAAACAAATGCAACAG-3′; second PCR: 5′-CACCATGATTTCATCGTCAGAGAT-3′ and 5′-TTAAGTTGTTGTTATAGCACAC-3′) and PrimeSTAR MAX DNA polymerase (Takara, Shiga, Japan) using cDNA derived from Col-0 rosettes. The amplified CDS was then cloned into a pENTR-D-TOPO vector (Thermo Fisher Scientific, Waltham, MA, USA). The AT2G32170.1 CDS was amplified in the same way using two primer sets (first PCR: 5′-ACCGAAGAGCCACCACC-3′ and 5′-CAGACACAAATAAAGAGAGTC-3′; second PCR: 5′-ATGGTTTCGCCGTCAGAGAGATG-3′ and 5′-TTAAGTTGTTGTTATAGCAC-3′). We attempted to clone the generated amplicon into a pCR8/GW/TOPO vector (Thermo Fisher Scientific); however, most of the resulting insertions were in the reverse direction, probably because the CDS in the desired orientation was toxic to *E. coli* strain DH5α. In addition, sequences cloned from amplicons inserted in the correct direction were complete aside from the introns. As an alternative, we first amplified the full-length vector sequence without introns using a cloned vector containing one intron in the correct direction as a template, PrimeSTAR MAX DNA polymerase (Takara), and primers for mutagenesis (5′-AATGATACACTGCCATGGGTCATGATT-3′ and 5′- TGGCAGTGTATCATTTTCATCCCAT-3′). Next, the Dpn I-treated amplified product was used to transform *E. coli*, resulting in the successful cloning of a vector with the CDS in the correct direction. Compared with non-transformed colonies, the colonies of bacteria carrying the vector in the correct direction were very small. The generated AT2G32160.3 (pENTR-D-TOPO) and AT2G32170.1 (pCR8/GW/TOPO) constructs were respectively introduced into overexpression vectors pASG-GW and pAKG-GW (Uchida et al., 2020) using Gateway LR Clonase II Enzyme mix (Thermo Fisher Scientific).

### GC-MS/MS analysis of MHs

For GC-MS/MS analysis, 100 μl aliquots of 250 μM πMH and τMH solutions were dispensed into separate 1.5 ml tubes. After evaporation of the solution using a centrifuge evaporator (SpeedVac, Thermo Fisher Scientific) and addition of 100 μl Mox regent (2% methoxyamine in pyridine, Thermo) to each tube, the metabolites were methoxylated at 30°C for approximately 6 h with shaking at 1,200 rpm using a thermo shaker (BSR-MSC100, Biomedical Sciences). Next, 50 μl of 1% (v/v) trimethylchlorosilane (TMS, Thermo Fisher Scientific) was added, and TMS derivatization was carried out by incubating the mixture at 37°C for 30 min with shaking at 1,200 rpm. Finally, 50-μl aliquots of the derivatized samples were dispensed into vials for GC-MS/MS analysis (AOC-5000 Plus with GCMS-TQ8040, Shimadzu). πMH-2TMS and τMH-2TMS were annotated using total ion chromatographs obtained by GC-MS/MS in scan mode, and MRM transitions (parent ion > daughter ion) were determined to be 218.00 > 73.10 for πMH-2TMS and 196.00 > 73.10 for τMH-2TMS. Plant extracts were derivatized in the same manner as used for πMH and τMH, and MHs in the extracts were analyzed by GC-MS/MS in MRM mode. Raw data collection was performed using GCMS Solution software (Shimadzu). GC-MS/MS parameters have been detailed previously (Tabeta et al., 2021).

### Expression analysis

The samples used for expression analysis were aerial parts of 3-week-old plants grown on agar-solidified half-strength MS medium (see the Plants section). Total RNA extraction, cDNA synthesis, and real-time PCR expression analysis were performed according to our previous study (Uchida et al., 2020). The primers used were as follows: 5′-TGATTGGTTGGATTCTTCGTTA-3′ and 5′-TTCCTTATTACCCAACGAACCTT-3′ for AT2G32160, 5′-GGTTGATGTAGATAAGGTTCGTTGT-3′ and 5′-AGGCTTGTAGCATTGATCTCG-3′ for AT2G32170, and 5′-GTTGGGATGAACCAGAAGGA-3′ and 5′-AAGAATACCTCTCTTGGATTGTGC-3′ for actin, the internal control.

## Results

### mGWAS and selection of novel candidate genes

We first conducted a preliminary metabolomic analysis based on LC-MS/MS using all *A. thaliana* seed accessions and selected 147 metabolites that were detected with high signal-to-noise ratios (> 3) and small relative standard deviation (< 10%) for a more detailed metabolomic analysis (**Supplementary Tables 4**–**9**). Next, we analyzed the metabolomes of seeds of 245 accessions and performed a GWAS of the metabolomic data to find associations (*p* < 1 × 10**^−^** ^5^, **Supplementary Figure 3**) between contents of 140 metabolites and SNPs (**Supplementary Table 10**). We then examined associations between metabolites and SNPs located in gene regions and found 1,683 candidate genes. As an example, the gene encoding methylthioalkylmalate synthase (MAM) showed associations with a number of glucosinolates with different side chains. In this study, we focused on AT2G32160, which was annotated as a methyltransferase gene and showed an association with 3-methyl-histidine (πMH), as methyltransferase was likely to be directly related to the biosynthesis of πMH, a methylated metabolite. Detected SNPs with low *p*-values (*p* < 1 × 10**^−^** ^9^) had no apparent effect on the gene function of AT2G32160 because they were located in introns or did not result in amino acid substitutions in exons. In contrast, SNPs with high *p*-values (*p* > 1 × 10**^−^** ^2^ in all batch) gave rise to nonsense mutations (i.e., TGG to TGA) (**Supplementary Table 11**).

The gene next to AT2G32160, AT2G32170, was annotated as a methyltransferase gene as well (**Supplementary Figure 4**). The functions of AT2G32160 and AT2G32170 have not been previously determined. In this study, we analyzed AT2G32170 in addition to AT2G32160 associated with πMH. For convenience, AT2G32160 and AT2G32170 are hereafter referred to as *MT160* and *MT170*, respectively.

### Expression analysis of *MT160* and *MT170*

**Figure 1:**
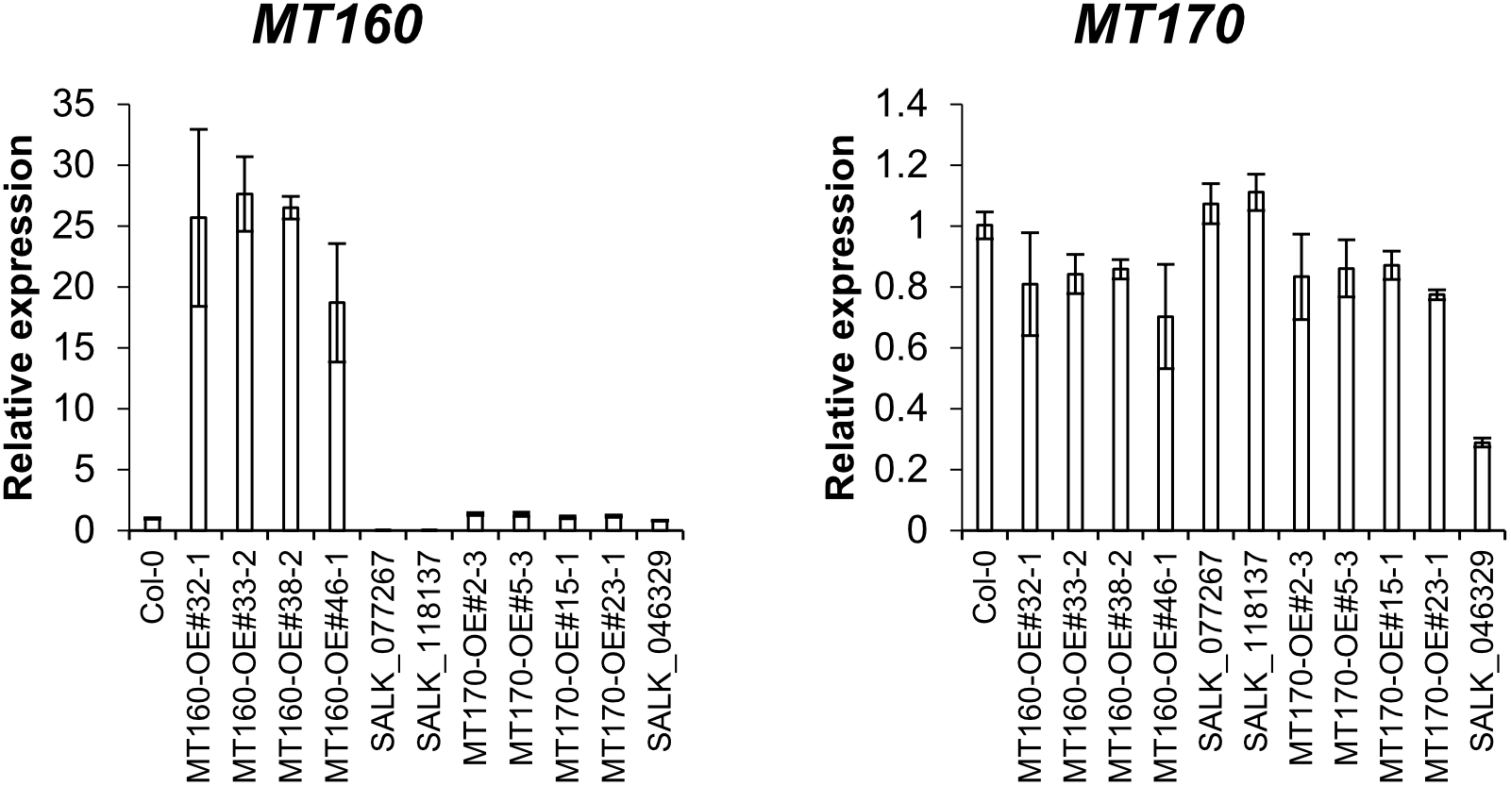
Expression analysis of *MT160* and *MT170* in aerial parts of 3-week-old *A. thaliana* plants based on real-time PCR. Bars indicate means ± standard error based on three biological replicates.

Expression levels of *MT160* and *MT170* in aerial parts of 3-week-old plants of the transgenic lines named *MT160*-OE and *MT170*-OE (see Materials and Methods) were analyzed by real-time PCR. According to the analysis, expression levels of *MT160* were increased by 19–28 fold in *MT160*-OE lines, whereas those of *MT170* were almost identical between *MT170*-OE lines and wild-type Col-0 (**Figure 1**). In addition, overexpression and disruption of *MT160* had no effect on *MT170* expression, and expression levels of *MT160* and *MT170* in SALK lines were respectively less than 1% and approximately 30% of those in Col-0.

### Structural analysis of MH in *A. thaliana* seeds by GC-MS/MS

**Figure 2:**
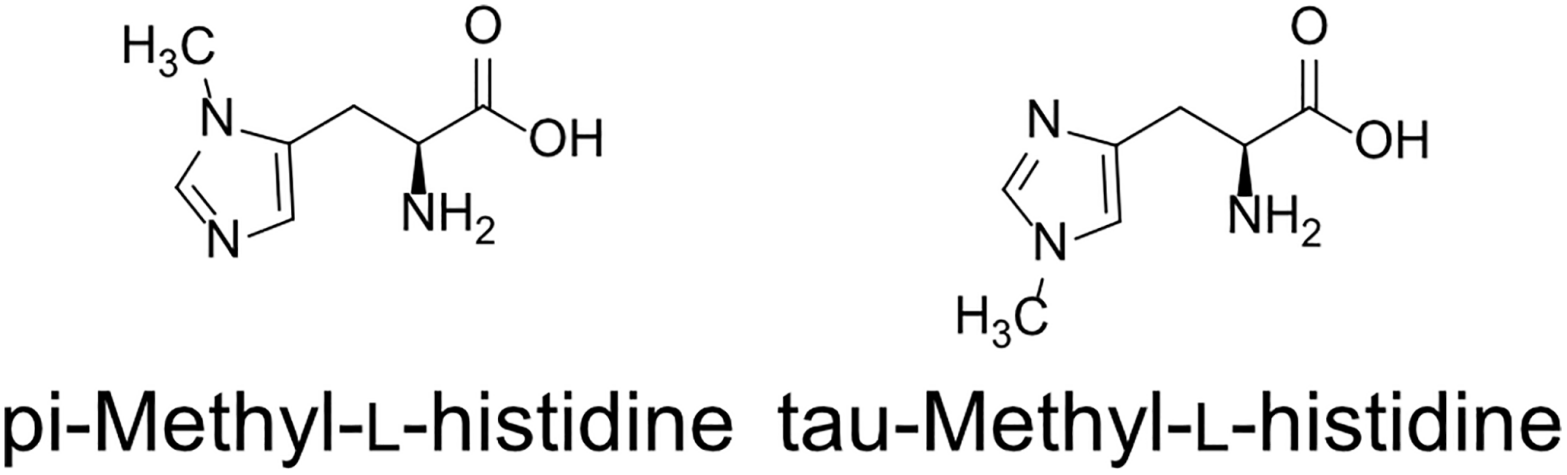
Structures of methylhistidine isomers.

**Figure 3:**
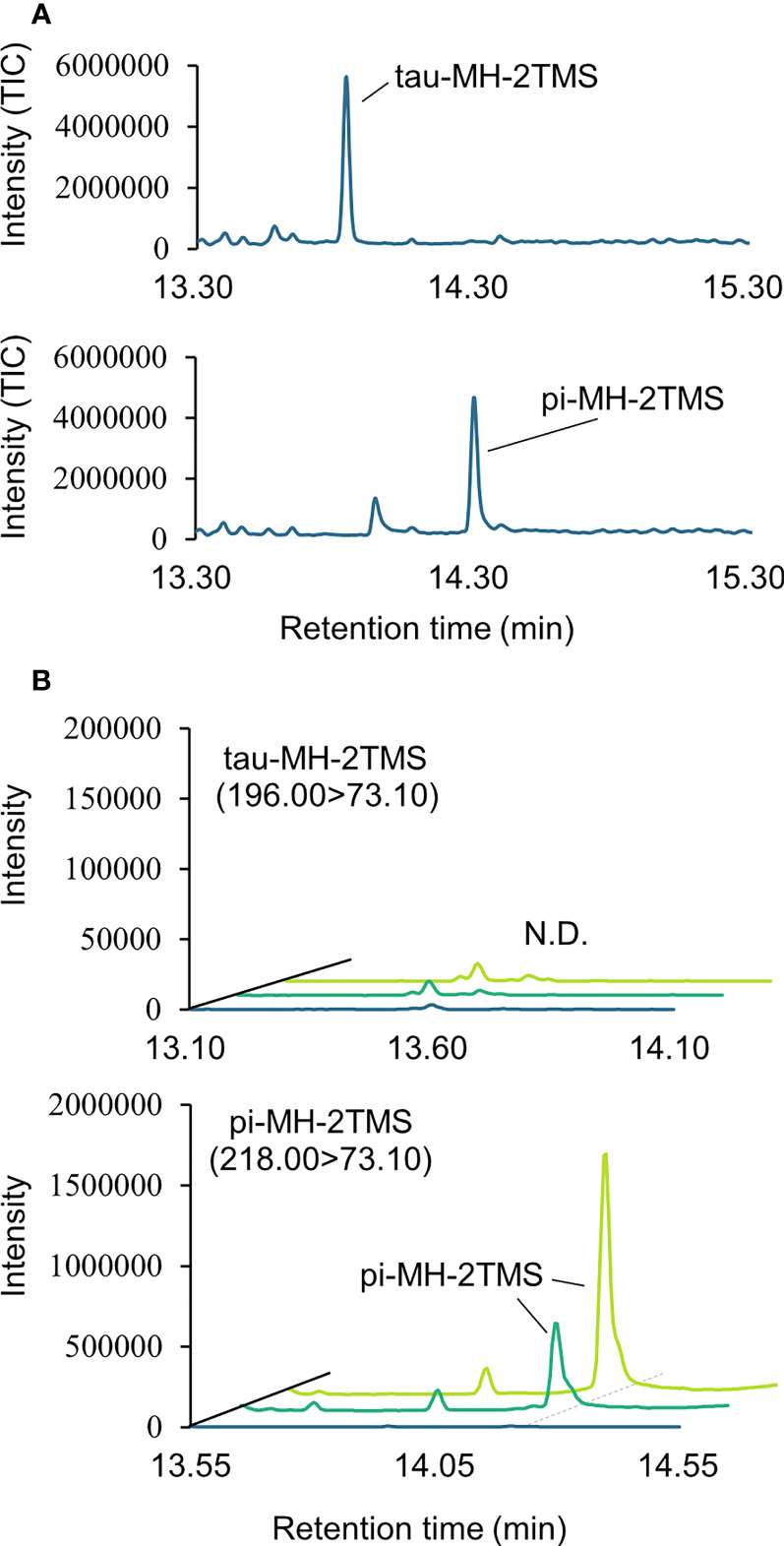
Determination of isomeric structures of methylhistidine (MH) in seeds of *Arabidopsis* lines by GC-MS/MS. **(A)** Chromatographs of τMH and πMH. Trimethylsilyl (TMS) standard samples were analyzed by GC-MS/MS in full scan mode. The total ion chromatograph (TIC) is indicated in blue. **(B)** Chromatographs obtained by GC-MS/MS in MRM mode. τMH-2TMS (196.00 > 73.10) and πMH-2TMS (218.00 > 73.10) were analyzed in the Col-0 (green), the overexpressing line *MT160*-OE#32-1 (lime yellow), and extraction buffer (blue). N.D., not detected.

Two types of MH, which differ in the position of the methyl group, have been found in living organisms: πMH and τMH (**Figure 2**). Because these two isomers could not be easily distinguished by our LC-MS/MS platform, the chemical structure of the metabolite detected in *A. thaliana* by LC-MS/MS was confirmed to be πMH by GC-MS/MS using TMS-derivatized extracts. The TMS-derivatized standard MH compounds were clearly separated in the GC-MS/MS chromatogram (**Figure 3A**). Comparison of the chromatograms of the seed extracts with those of the two MH standards revealed that the peak corresponding to πMH-2TMS (m/z 218.00 > 73.10), but not that corresponding to τMH-2TMS (m/z 196.00 > 73.10), was present in all samples (**Figure 3B**). This result confirms that the metabolite detected by LC-MS/MS was actually πMH.

### Quantification of πMH in *MT160*- and *MT170*-OE and knockout lines

**Figure 4:**
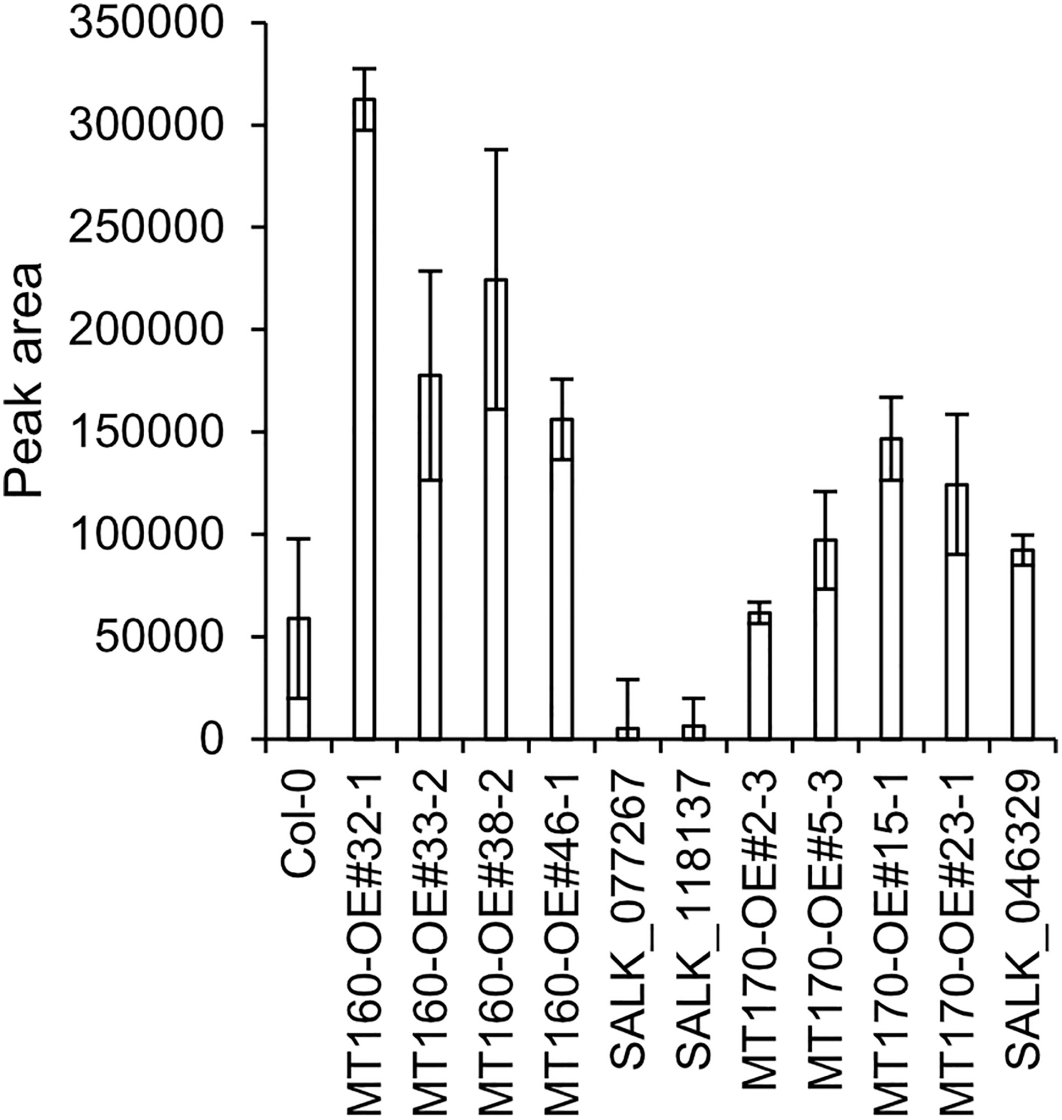
πMH content of seeds of *Arabidopsis* lines. Bars indicate means ± standard error based on three replicates, each comprising approximately 50 seeds.

**Figure 5:**
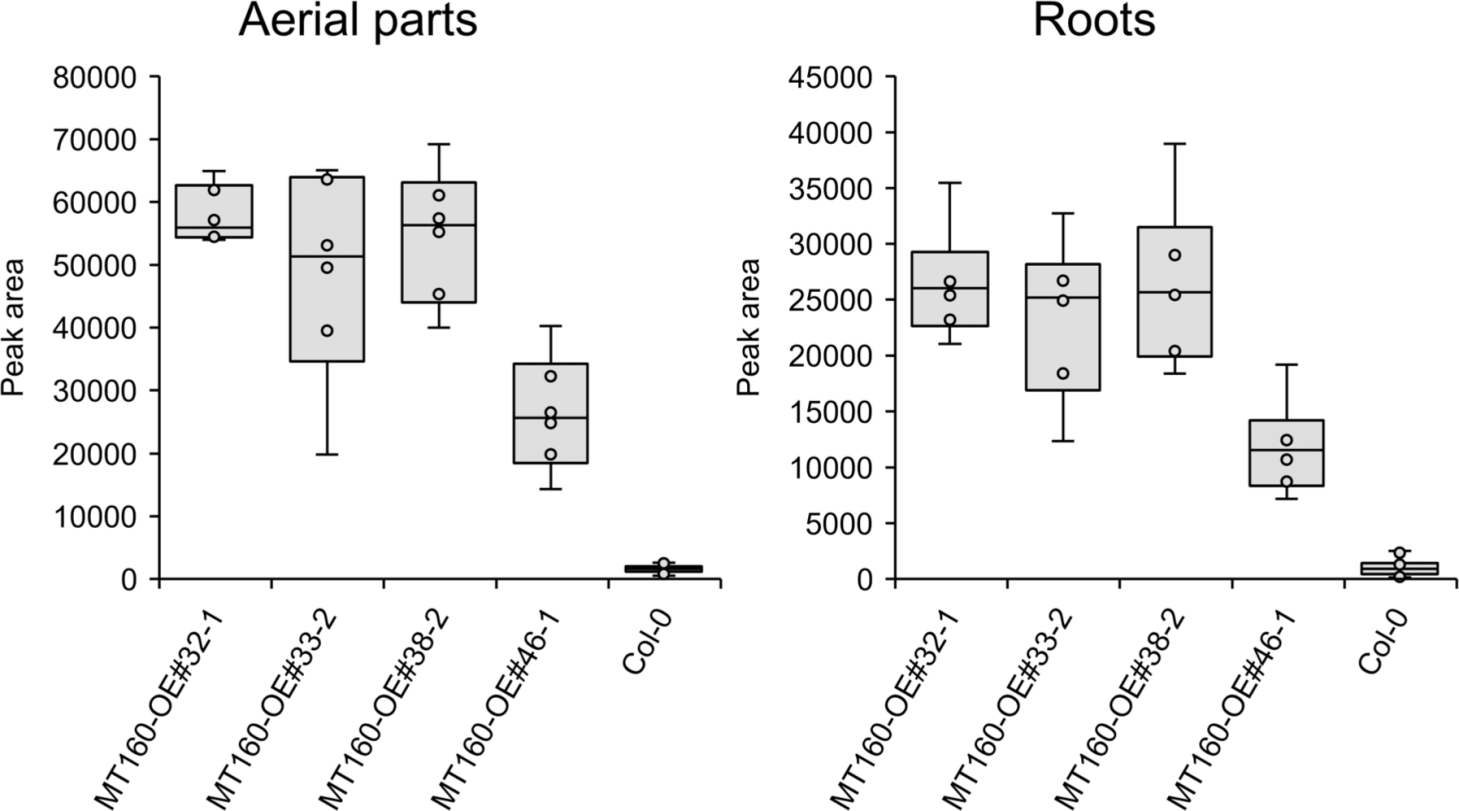
Box plots of the πMH content of aerial parts and roots of *MT160*-OE lines and the Col-0 wild type. The number of biological replicates is as follows: *n* = 6 (*MT160*-OEs); *n* = 24 (Col-0; four independent experiments with six individuals each).

πMH contents of seeds of *MT160*-OE and -knockout lines were respectively approximately 3.7-fold higher and one-tenth lower than those of Col-0 (**Figure 4**; **Supplementary Table 12**). Although expression levels of *MT170* in *MT170*-OE lines were unchanged relative to those in Col-0, seeds of some lines had increased πMH contents (**Figure 5**; **Supplementary Table 12**). The amount of πMH in aerial parts and roots of hydroponically grown plants was increased by 20–35-fold and 12–28-fold, respectively, in *MT160*-OE lines compared with Col-0 (**Figure 5**; **Supplementary Table 13**). In contrast, πMH contents of *MT160*- and *MT170*-knockout lines and *MT170*-OE lines were unchanged (**Supplementary Figure 5**; **Supplementary Tables 14**, **15**).

## Discussion

In this study, we conducted a completely data-driven mGWAS using *A*. *thaliana* seeds without any preliminary information. We detected many SNPs associated with the accumulation of various metabolites and found associations between aliphatic glucosinolate compounds and SNPs on MAM genes. Given that MAMs are essential enzymes for the side-chain elongation of aliphatic glucosinolates, these uncovered associations can be considered to constitute a positive control for the mGWAS and thus validate the results of our study. As a target for further investigation, we focused on a methyltransferase gene of unknown function showing an association with πMH accumulation. Because *MT160* is an enzyme gene, we expected nonsense mutations to have the highest impact on πMH content. We discovered, however, πMH contents of accessions carrying the nonsense mutation were not significantly different from those in accessions harboring other SNPs, and therefore the *p*-value of the SNP causing the nonsense mutation was higher than that of SNPs located in introns or those responsible for silent mutations (**Supplementary Table 11**; **Supplementary Figure 6**). The absence of a significant influence on the phenotype, despite nonsense mutations detected, could potentially be attributed to factors such as latent redundant genes that bypass such nonfunctional mutations, or mechanisms like stop codon read-through, which suppresses the termination of translation. The TGA (UGA) codon, also generated by the nonsense mutation of *MT160* gene, is reported to be the stop codon most prone to read-through in recent studies in *A. thaliana* (Xu et al., 2020; Sahoo et al., 2022). Regarding the candidates of the causative mutation that could impact πMH content, our study, which utilized polymorphism data derived from the 250K SNP-Chip, could not rule out the possibility of undetectable polymorphisms in the *MT160* region. In this study, we demonstrated that our mGWAS-based approach identified the gene encoding methyltransferase, a key enzyme for *N*-methylhistidine biosynthesis in *A. thaliana*, while our results also illustrated the challenges of identifying causative mutations for the metabolic phenotypes observed in the population. Alongside ensuring exhaustive coverage of genetic variations within the population, efforts towards exploring genes that could potentially bypass metabolic networks, as well as enriching the annotation for polymorphic sites—including those involving potential stop codon read-through—may prove beneficial in further elucidating the genetic basis of metabolic diversity in *A. thaliana*.

Histidine methyltransferases have been found in yeast (Hpma1p or YIL110W) and mammals (SETD3, UPF0586, and METTL9) and respectively methylate the π- or τ-position of histidine (Webb et al., 2010; Kwiatkowski et al., 2018; Wilkinson et al., 2019; Davydova et al., 2021). Although amino acid sequence identity among YIL110W (yeast), UPF0586 (rat), and MT160 is low, all of these proteins have an N-2227 domain (Drozak et al., 2015). In our study, overexpression of *MT160* increased the content of histidine methylated at the π-, but not τ-, position, similar to YIL110W and UPF0586. All enzymes with an N-2227 domain are thus considered to methylate the pi-position of histidine in all species, and MT160 most likely functions as a histidine *N*_π_-methyltransferase. Given that πMH content respectively increased and decreased in *MT160*-OE and knockout lines in our study, MT160 certainly appears to be involved in πMH biosynthesis in *A. thaliana*.

Methylated amino acids have been found in various plant species (Eloff, 1980; Fourré and Lhoest, 1989; Tyihák et al., 1989; Waterborg, 1993). To our knowledge, however, MH has only been reported in calli of barley and seashore paspalum (*Paspalum vaginatum*) (Katoh, 1990; Shi et al., 2020). In the present study, recombinant protein of MT160 could not be purified because the protein was not produced in the *E. coli* expression system (data not shown). The detailed enzymatic properties of MT160 are thus still unknown, and whether the target molecule of MT160 is free histidine or a histidine residue within proteins is unclear. Future work is needed to identify the target molecule of MT160.

In contrast to *MT160*, disruption of *MT170* did not affect plant MH contents. In addition, *MT170*-OE lines did not overexpress *MT170* for some unknown reason. One possible reason is that overexpression of *MT170* may have negatively affected *Arabidopsis* growth and thus overexpressing line could not be obtained. Moreover, a small amount of πMH was detected in *MT160*-knockout lines and some accessions with nonsense mutations in the *MT160* gene. These facts suggest that other factors besides *MT160* and *MT170* are involved in the variation in πMH content in response to environmental conditions. Furthermore, genes of unknown function harboring SNPs very strongly associated with πMH content were found on chromosome 5 (**Supplementary Figure 3**; **Supplementary Table 10**) and may have an effect on πMH content.

The significance of methylation of free histidine or histidine residues in proteins is currently unclear. According to a recent study, METTL9 may participate in mammalian immune response by pi-methylating the proinflammatory protein S100A9 (Daitoku et al., 2021; Davydova et al., 2021), and METTL18 regulates the rate of translation by tau-methylating histidines in ribosomes (Matsuura-Suzuki et al., 2022). In plants, however, the function of πMH has not been clarified. Our study findings thus provide useful new insights into the role of histidine methylation in plants.

## Data availability statement

The original contributions presented in the study are included in the article/**Supplementary Material**. Further inquiries can be directed to the corresponding authors.

## Author contributions

KU generated the overexpression lines and carried out the expression analysis. J-SK and KM performed the GWAS. MS conducted the metabolomic analysis. HT carried out the GC-MS analysis. KU and MH wrote the manuscript. All authors contributed to the article and approved the submitted version.
