# Supplementary material for: A metabolome genome-wide association study implicates histidine N-pi-methyltransferase as a key enzyme in N-methylhistidine biosynthesis in Arabidopsis thaliana
Source: Front Plant Sci. 2023 Jun 8;14:1201129. doi: 10.3389/fpls.2023.1201129 (PMC10285387; doi:10.3389/fpls.2023.1201129)
Supplement: Supplementary file 1 [file DataSheet_1.pdf]

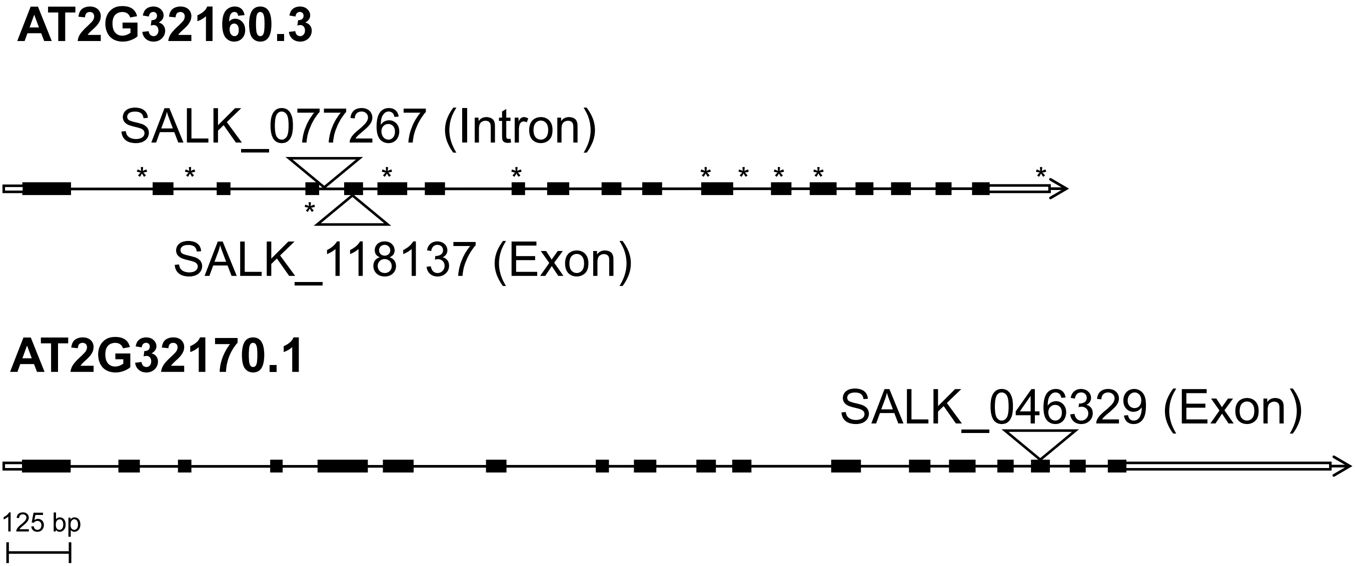

Supplementary Figure 1
Schematics diagrams of the two candidate genes.
Boxes and lines indicate exons and introns, respectively. Asterisks indicate approximate SNP locations.

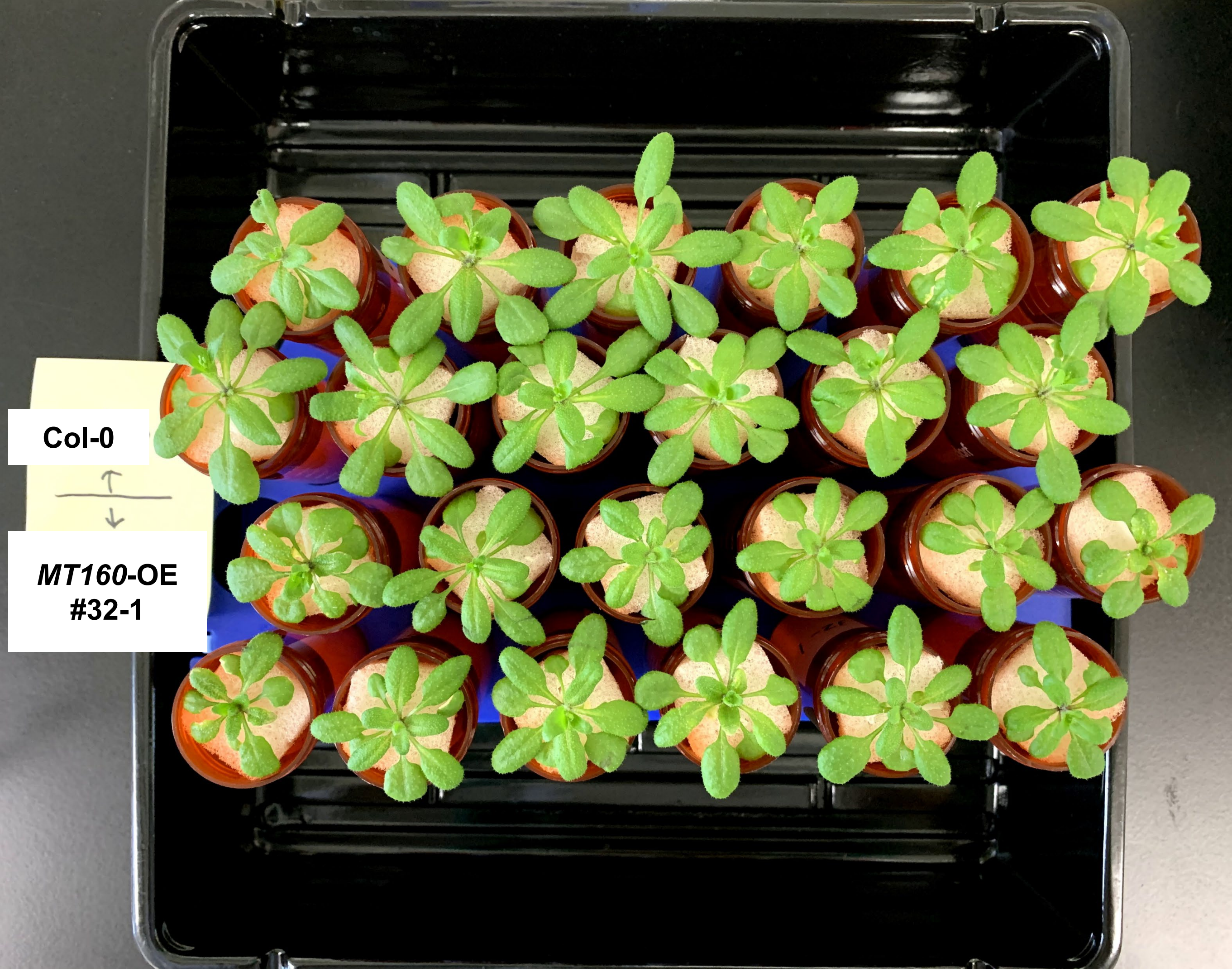

Supplementary Figure 2
Arabidopsis thaliana plants hydroponically cultured in 50-ml tubes.
Upper half, Col-0; lower half, *MT160*-OE#32-1

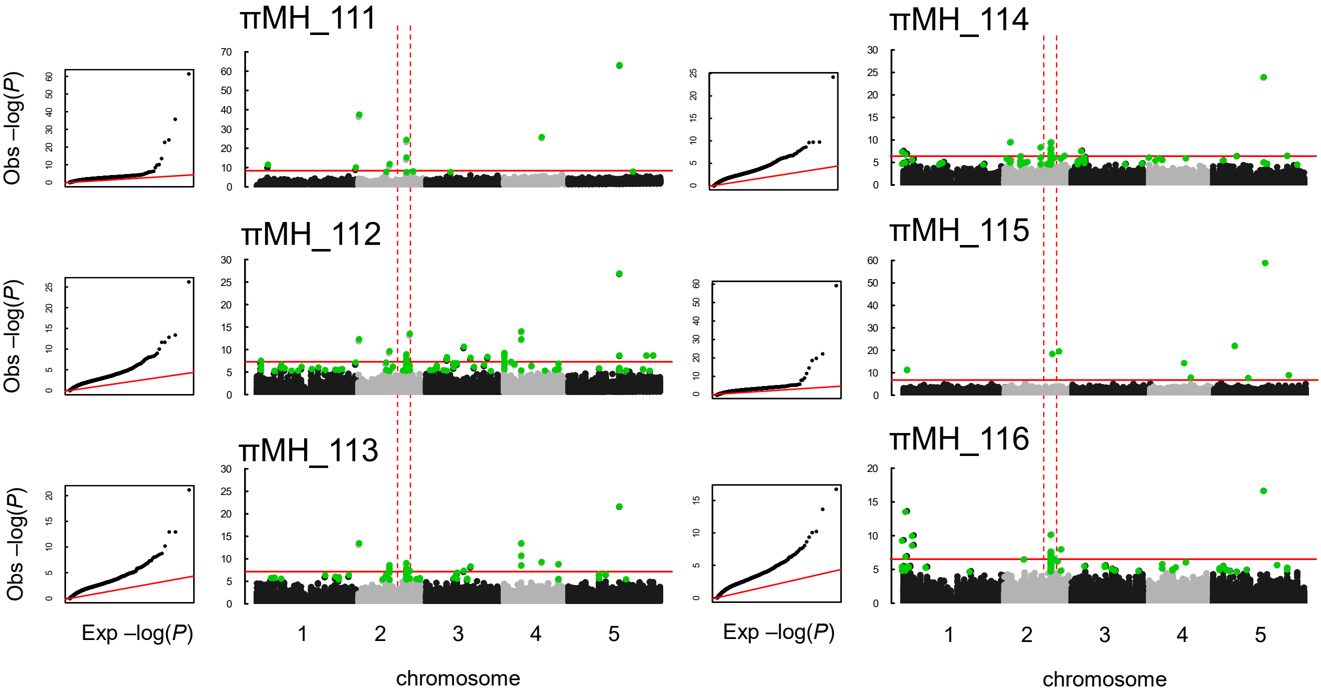

Supplementary Figure 3 Genome-wide associations between πMH and SNPs based on six metabolomic datasets (#111–#116) of *Arabidopsis* seeds.

Quantile–quantile plots and Manhattan plots are respectively shown on the left and right side of each panel. Vertical and horizontal axes indicate *p*-values and chromosomal positions, respectively.

Red horizontal lines in Manhattan plots indicate the Bonferroni threshold.

Green dots indicate statistically significant SNPs (FDR < 0.05).

Vertical dotted lines delimit the location of the SNP near *AT2G32160*.

Metabolomic datasets #111–#113 and #114–#116 are comprised of data from 245 accessions and that from 235 accessions , respectively.

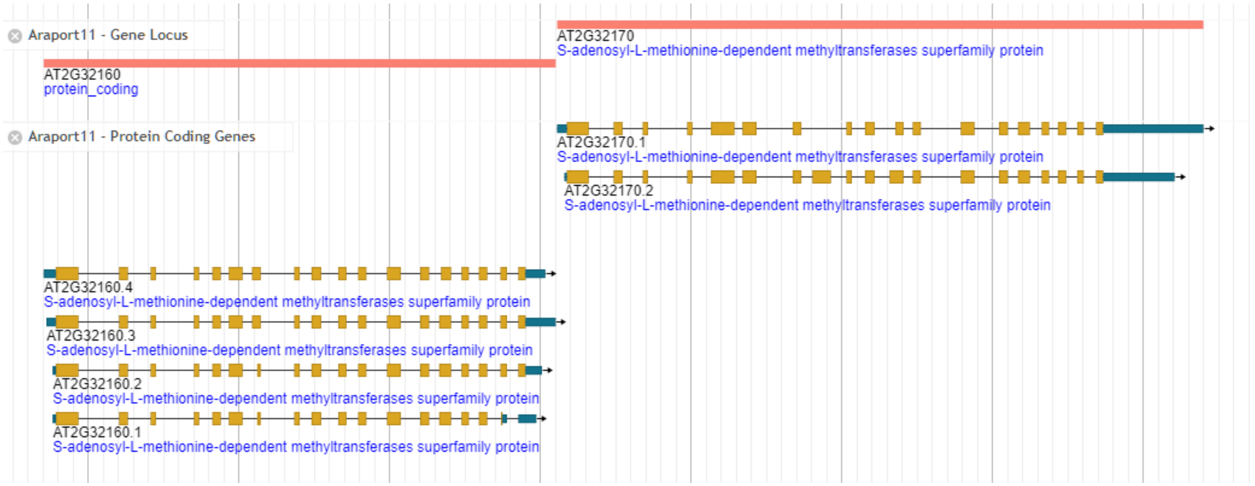

Supplementary Figure 4

Genomic locations of AT2G32160 and AT2G32170.

Images were obtained from JBrowse (https://jbrowse.arabidopsis.org/)

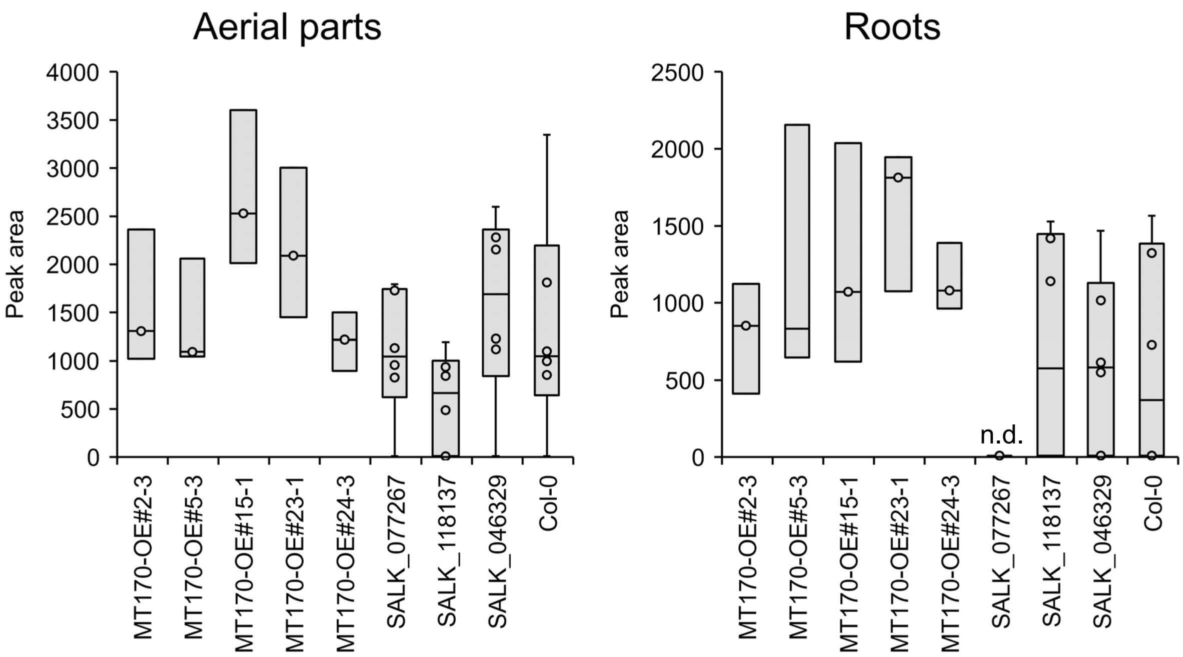

Supplementary Figure 5
Box plots of the πMH content of aerial parts and roots of *MT170*-OE and SALK Arabidopsis lines. The number of biological replicates is as follows: *n* = 3 (*MT170*-OEs); *n* = 6 (SALK lines and Col-0).

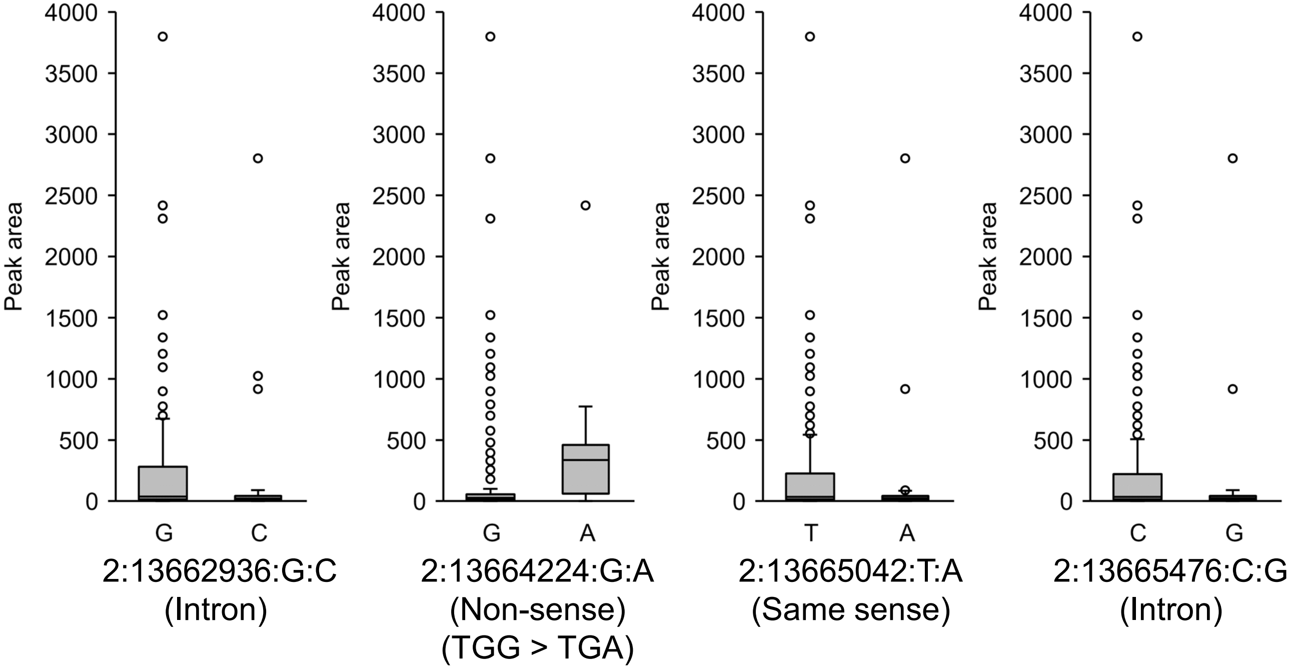

Supplementary Figure 6 Boxplots of πMH content by SNP type from metabolomic dataset #114.
